# Traumatic Pneumothorax Following Acupuncture: A Case Series

**DOI:** 10.5811/cpcem.2016.11.32757

**Published:** 2017-01-23

**Authors:** Felix Grusche, Diana Egerton-Warburton

**Affiliations:** * Monash University, Melbourne, Victoria, Australia; † Monash Medical Centre, Department of Emergency Medicine, Clayton, Victoria, Australia

## Abstract

Acupuncture and dry needling are used by a range of health professionals to treat conditions such as musculoskeletal pain. Treatment occurs both in an outpatient setting and in emergency departments (ED). Acupuncture and dry needling are considered to be relatively safe techniques with a low risk of serious adverse events. We report three cases of traumatic pneumothorax following acupuncture/dry needling that presented to our ED between 2014 and 2016.

## INTRODUCTION

Acupuncture is a popular alternative medicine technique that has been practiced in China for over 3,000 years. It involves the placement of solid needles of 10 – 100 mm length through the skin. Depth of insertion varies from a few mm to several cm, often into muscle tissue. For musculoskeletal (MSK) conditions, needles of 0.2 – 0.35 mm diameter are typically placed 10 – 65 mm deep into tissue and left *in situ* for 10–30 min.^1^ It has been estimated from post-mortem examinations that a puncture depth of 10 – 20 mm is sufficient to reach the lungs.^2^ We report three cases of traumatic pneumothorax following acupuncture/dry needling that presented to our hospital. All cases involved young females of low body mass index (BMI) who underwent acupuncture/dry needling to the shoulder and upper back region of shoulder for MSK pain.

## CASE REPORTS

### Case 1

A 24-year-old female with a BMI of 22 had dry needling to her left posterior shoulder for MSK pain; 30 minutes later she developed severe pain around the site and shortness of breath. She presented to the ED five hours later with a respiration rate of 22 breaths per minute (BPM) and SpO_2_ of 98% on room air. Her pain score was 7/10 on a numeric rating scale (NRS). Reduced air entry over the left anterior chest was noted by auscultation. A chest radiograph (CXR) revealed a 22% left apical pneumothorax (calculated by interpleural distance).^3^ The patient was observed overnight and discharged after a repeat CXR the following morning showed no change of the pneumothorax size. A repeat CXR 10 days later showed full resolution.

### Case 2

A 21-year-old female with a BMI of 18 developed severe, right-sided chest pain and shortness of breath shortly after receiving acupuncture for “knots” in her neck. She presented to the ED the following day with 7/10 NRS right-sided pleuritic chest pain. Her vital signs were a heart rate of 102, a respiration rate of 22 BPM and SpO_2_ of 100% on room air. Decreased breath sounds were noted over the right upper zone. A CXR demonstrated a 26.3% right-sided pneumothorax.^3^ The patient was discharged home and re-presented the following day for a repeat CXR, which demonstrated no change in pneumothorax size. On day four after her initial presentation, the patient re-presented with sudden onset of worsening shortness of breath and pain. A CXR was taken and showed that the pneumothorax was resolving. She was given stronger analgesia, which improved her symptoms. A further repeat CXR at nine days showed full resolution of the pneumothorax.

### Case 3

A 21-year-old female with a BMI of 21 presented with 6/10 (NRS) sharp left-sided chest pain, dizziness, nausea and pre-syncope one-and-a-half hours after dry needling for left-sided MSK shoulder pain. Her vital signs were a heart rate of 66, a respiration rate of 24 BPM and SpO_2_ of 100% on room air. Decreased breath sounds were noted over the left lung field. A CXR demonstrated a 16.2% left apical pneumothorax.^3^ A repeat CXR four hours later showed a 20.6% pneumothorax.^3^ Since the patient remained clinically stable, she was managed conservatively with a follow-up appointment three days later. Here, CXR showed that the pneumothorax was resolving. At a further appointment 21 days after the initial presentation, CXR demonstrated full resolution.

## DISCUSSION

Health professionals including ED clinicians use acupuncture/dry needling.^4^ It is generally considered to be low risk. Serious complications such as pneumothorax, subarachnoid hemorrhage, nerve lesions, infection and retained needles have been reported in the literature but are thought to be rare.^5^,^6^ A large prospective observational study assessing the safety of acupuncture in Germany 7 and a review of safety data from the British National Health Service found an incidence of pneumothorax of less than one in a million per procedure.^8^,^9^ Since these studies included all applications of acupuncture, for example including to the knee, the incidence of pneumothorax due to acupuncture to the chest wall is likely to be higher. Our report of three cases in as many years supports that notion. Since all cases occurred in young women of low BMI we further hypothesize that this patient population is at increased risk.

## CONCLUSION

Traumatic pneumothorax is an important differential in the setting of dyspnea with recent acupuncture/dry needling. Patients with a low BMI may be at particular risk. Furthermore, patients should be informed of this uncommon but serious complication before undergoing acupuncture/dry needling.
